# Network toxicology based research into the effect and mechanism of plasticizers on infertility

**DOI:** 10.3389/fphar.2026.1811097

**Published:** 2026-07-14

**Authors:** Wenxiang Wang, Yafang Zhang, Mengyuan Chang, Wenqiang Fan, Yufei Qin, Zhanghuan Li, Xiangyi Shen, Yanli Liu

**Affiliations:** 1 Department of Gynecological Oncology, Xinxiang Central Hospital, The Fourth Affiliated Hospital of Henan Medical University, Xinxiang, Henan, China; 2 Stem Cell and Biotherapy Technology Research Center, School of Life Science and Technology, Henan Medical University, Xinxiang, China; 3 Department of Rheumatology and Immunology, Xinxiang Central Hospital, The Fourth Affiliated Hospital of Henan Medical University, Xinxiang, Henan, China; 4 Henan Key Laboratory of Medical Tissue Regeneration, Henan Medical University, Xinxiang, China; 5 Henan Joint International Research Laboratory of Stem Cell Medicine, School of Biomedical Engineering, Henan Medical University, Xinxiang, China

**Keywords:** infertility, mechanism, molecular docking, network toxicology, plasticizer

## Abstract

**Introduction:**

To investigate the potential impact of plasticizers on infertility and elucidate the underlying mechanisms, we employed an integrated approach combining network toxicology, molecular docking, and in vitro experimental validation.

**Methods and results:**

Initially, we identified potential targets by intersecting plasticizer-related targets with infertility-associated targets, yielding 134 candidate targets. These targets were subsequently analyzed using the STRING database and Cytoscape software to construct protein-protein interaction (PPI) networks, from which 20 hub genes were identified. Functional enrichment analysis conducted using the R software revealed that these targets participate in multiple biological pathways. Molecular docking simulations performed with AutoDock Vina and visualized using PyMol demonstrated strong binding affinities between key hub targets and three representative plasticizers: diethyl phthalate (DEP), dimethyl phthalate (DMP), and dioctyl phthalate (DOP). For experimental validation, we examined the effects of plasticizers on the human ovarian granulosa cell lines. CCK-8 assays revealed significant inhibition of cell proliferation following exposure to plasticizer. Flow cytometry and western blot analyses further showed that plasticizer treatment upregulated the anti-apoptotic protein BCL-2 while downregulating pro-apoptotic BAX, as well as key reproductive markers, including CYP19A1, ER-α, and FSHR.

**Discussion:**

Collectively, these findings demonstrate that plasticizers may contribute to infertility through multiple molecular pathways, as predicted by network toxicology and confirmed by in vitro experiments. This integrated approach provides compelling evidence for the reproductive toxicity of plasticizers and offers insights into their potential mechanisms of action.

## Introduction

Plasticizers are a class of chemical additives extensively employed to enhance the flexibility and processability of plastic materials (Dalamaga et al., 2024). Diethyl phthalate (DEP), dimethyl phthalate (DMP), and dioctyl phthalate (DOP) are the most prevalent compounds in this category and are ubiquitously found in consumer products, ranging from personal care items and children’s toys to medical equipment and food packaging (Zhang et al., 2024). Although their lipophilic nature and environmental stability contribute to their functional utility, these properties promote environmental persistence and bioaccumulation in both ecosystems and human tissues. This dual characteristic has sparked significant scientific concern regarding their potential as endocrine disruptors and their implications for long-term human health (Patisaul and Adewale, 2009).

Infertility, a disorder of the female reproductive system, is clinically defined as the inability to achieve pregnancy after 12 months of regular unprotected intercourse (Vakili and Jafarinia, 2024). This condition arises from the complex interplay between genetic predisposition, lifestyle factors, and environmental exposure (Vakili and Jafarinia, 2024). Notably, the increasing global prevalence of infertility has been partially linked to environmental contaminants, with growing concerns over the role of prolonged exposure to endocrine-disrupting chemicals (EDCs), including plasticizers (Madbouly et al., 2012). Although extensive research has demonstrated that certain environmental chemicals contribute to impaired fertility, the specific impact of plasticizers on infertility pathogenesis remains underexplored, warranting further investigation.

To address this emerging health concern, our study employed an integrated computational approach to systematically examine the molecular interactions between prevalent plasticizers (DEP, DMP, and DOP) and infertility-related proteins. By combining network toxicology with molecular docking simulations, we aimed to uncover the potential mechanisms by which these compounds may contribute to reproductive dysfunction. Based on systems biology theory, network toxicology offers a new strategy for exploring the relationship between molecules and diseases by integrating systems biology, multidirectional pharmaceutical biology, bioinformatics, and computer science. Network toxicology has shifted the study of biological systems from a single-drug, single-target to a multi-drug, multi-target paradigm (Chen and Hou, 2024),Network toxicology, an interdisciplinary methodology bridging bioinformatics, systems biology, and cheminformatics, offers a system-level perspective to identify how these chemicals may perturb critical biomolecular networks and cellular processes, potentially leading to pathological outcomes (Chen and Hou, 2024). Complementing this approach, molecular docking provides an atomic-level resolution of the binding interactions between plasticizers and target proteins, offering structural insights into their potential endocrine-disrupting activities.

## Methods and materials

**FIGURE 1:**
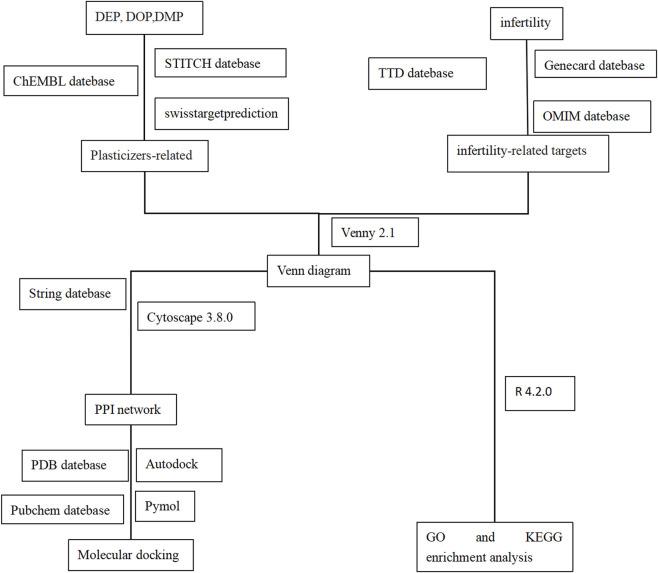
Process flow diagram of the study of plasticisers in the treatment of Infertility.

The flow chart of the study design was shown in Figure 1.

### Collection of potential targets of DEP, DMP, and DOP

The active targets of diethyl phthalate (DEP), dimethyl phthalate (DMP), and dioctyl phthalate (DOP) were comprehensively identified using three established bioinformatics resources: the ChEMBL database (https://www.ebi.ac.uk/chembl/), STITCH database (http://stitch.embl.de/), and SwissTargetPrediction platform (http://www.swisstargetprediction.ch/). ChEMBL represents a manually curated repository of bioactive molecules with drug-like properties that integrates chemical, bioactivity, and genomic data to facilitate drug discovery and development. STITCH serves as an interaction exploration platform, documenting both experimentally validated and computationally predicted associations between chemicals and proteins, with evidence derived from multiple sources including experimental data, literature reports, and existing databases. SwissTargetPrediction provides a robust computational framework for predicting the molecular targets of bioactive compounds in humans and other vertebrates, offering valuable insights into the potential mechanisms of action, off-target effects, and side-effect profiles of small molecules.

### Collection of potential targets for infertility

Human infertility-associated genes were systematically retrieved from three authoritative databases: the Therapeutic Target Database (TTD) (http://db.idrblab.net/ttd/), OMIM (Online Mendelian Inheritance in Man) (OMIM; https://www.omim.org/), and GeneCards (https://www.genecards.org/). TTD catalogs experimentally validated and explored therapeutic targets, including proteins and nucleic acids, along with their associated diseases, pathways, and corresponding drugs, The selection criteria are genes that have been successfully validated. OMIM serves as a comprehensive knowledgebase of human genes and genetic disorders and is widely utilized by researchers and clinicians to elucidate gene-disease relationships. GeneCards is an integrative platform that aggregates gene-disease associations from over 150 web sources, providing consolidated annotations on gene function and relevance to human pathologies, The filtering criterion is “Protein-coding”. Using the keyword “infertility”, we queried these databases to extract genes linked to infertility, including their official names and gene identifiers. The retrieved gene sets from all three sources were then merged and duplicate entries were removed to generate a non-redundant gene list for subsequent analyses.

### Constructing protein-protein interaction network

To identify potential interactions between phthalate-associated targets (DEP, DMP, DOP) and infertility-related genes, we first intersected the two gene sets using the Venn diagram tool in the Venny 2.1 online platform (https://bioinfogp.cnb.csic.es/tools/venny/). Overlapping genes representing shared targets between phthalate exposure and infertility were extracted for further analysis. Next, the overlapping gene list was submitted to the STRING database (https://cn.string-db.org/) to construct a protein-protein interaction (PPI) network. The search was restricted to *Homo sapiens*, with an interaction confidence score threshold of ≥0.7 to ensure high reliability. The resulting PPI network was then imported into Cytoscape (v3.9.1) via the STRING application for network visualization and topological analysis.

### Topological analysis and core gene identification

The topological properties of each node in the PPI network were quantitatively analyzed using Cytoscape software (version 3.8.0). Through a comprehensive network analysis, we calculated multiple centrality metrics, including degree centrality, betweenness centrality, and closeness centrality, to evaluate the relative importance of each node in the network. Based on these integrated topological scores, we systematically identified the top 20 highest-scoring core genes that might play pivotal roles in the potential therapeutic effects of DEP, DMP, and DOP on infertility. These high-priority candidate genes were selected for further in-depth analysis.

### Functional enrichment analysis of candidate targets

To elucidate the potential therapeutic mechanisms of DEP, DMP, and DOP in infertility treatment, we conducted a comprehensive Gene Ontology (GO) enrichment analysis of the identified target genes. This analysis systematically evaluated the biological relevance of these targets across three fundamental categories: biological process (BP), cellular component (CC), and molecular function (MF). The analysis was performed using R package ClusterProfiler (version x. y.z) from Bioconductor, implemented in R x64 4.0.3. ClusterProfiler is a robust bioinformatics tool specifically designed for the functional enrichment analysis of gene clusters and offers advanced statistical methods for interpreting high-throughput genomic data.

### Molecular docking stimulation

Autodock is the leading software for molecular docking to determine the plausibility of target genes, and the branch software Autodock 4 and Autodock Vina were used in this study. In addition, Pymol software, a sub-software of Python, was used to process and analyze the molecular docking results. Chem 3D software is typically used to analyze and process the chemical molecular structures. The 2D structure of the active component was downloaded from PubChem (https://pubchem.ncbi.nlm.nih.gov/) and the 3D structure of the target protein was downloaded from the PDB online platform (https://www.rcsb.org/). The 2D structure of the obtained active component was imported into chem3D software to generate a 3D structure with minimal energy states, and all the obtained 3D structures were imported into Autodock 4 software for dehydration and hydrogenation. The results were then subjected to bulk molecular docking using AutoDock Vina and Open Babel based on the principle of semi-flexible molecular docking. The results were then subjected to bulk molecular docking using AutoDock Vina and Open Babel based on the principle of semi-flexible molecular docking. Energy < -7.0 kcal/mol was used as a criterion for screening in the analyzed results, The top 20 most tightly connected docking results were screened and imported into Pymol software for further processing, analysis, and generation images showing the bond and bond length between the molecule and the target protein.

### Cell culture

The SVOG and KGN human ovarian granulosa cell lines were purchased from OTWO Biotechnology Co., Ltd. (HTX2650, HTX 2045, China) and cultured in high-glucose DMEM (ZQ-101; ZQXZBIO, China) containing 10% (v/v) fetal bovine serum and 1% (v/v) penicillin/streptomycin at 37 °C with 5% CO2. When the cells reached 90% confluence, they were subcultured. Both SVOG and KGN cells were authenticated by STR profiling.

### CCK8 assay

Cells were seeded in a 96-well plate (5 × 103 cells/well) and cultured overnight. Then, the storage solution of MEHP (Aladdin, China) (final concentrations: 0, 0.2, 0.4, 0.6, 0.8, 1.0, 1.2, and 1.4 mM) was added to each well, and the cells were cultured for another 24 h. Then, 5 μL of CCK-8 solution (Beyotime, C0038, China) was added to each well, and after incubation for 3 h, the absorbance at 450 nm was determined using a microplate reader (Thermo, United States).

### Apoptosis assay

The SVOG were treated with 0.8, 1.0, 1.2 mM of MEHP and the KGN were treated with 0.6, 0.8, 1.0 mM of MEHP for 24 h, and then the cells were digested and gently re-suspended in a staining buffer containing 5 μL Annexin V-FITC storing solution and 10 μL storing propidium iodide solution according to the instructions of Annexin V-FITC detection Kit (Beyotime, China); after that, the cells were incubated for 20 min in the dark at 4 °C. At the end of the incubation period, the cells were detected using flow cytometry (Agilent, United States of America).

### Calcein AM/PI assay

The cells were treated with different concentrations of MEHP for 24 h. After removing the culture medium, 250 μL of Calcein AM/PI assay working solution (Beyotime, C2015S, China) was added to each well and incubated at 37 °C in the dark for 30 min. The cells were observed and imaged using an inverted fluorescence microscope (Leica, Germany).

### Western blot

SVOG was treated with 0.8, 1.0, 1.2 mM of MEHP and KGN was treated with 0.6, 0.8, 1.0 mM of MEHP for 24 h. After treatment, protein samples of harvested cells were prepared using RIPA Lysis Buffer (Beyotime, China). Proteins were separated by SDS-PAGE before being transferred to polyvinylidene difluoride membranes (Millipore), and the membrane with targeted proteins was blocked for 1 h at room temperature in 5% nonfat milk, followed by overnight incubation with primary antibody at 4 °C. After washing with TBST three times, the membranes with the targeted proteins were incubated with the secondary antibody for 1 h at room temperature. Finally, the signals were visualized using the ECL Iuminescence reagent (Beyotime, China) and detected using an Amersham Imager 800 (GE Healthcare Life Sciences, United States) with ImageJ software.

### Statistical analysis

Statistical analysis of the bioinformatics data and mapping was performed by R x64 4.0.3, Cytoscape 3.8.0, and PyMOL software. The experimental data from this study were quantitatively analysed using ImageJ software, and the figures were produced using GraphPad Prism and Adobe Photoshop CS6. Comparisons between two groups were analysed using an independent samples t-test, whilst comparisons involving three or more groups were analysed using one-way analysis of variance (ANOVA). A p-value of <0.05 was considered statistically significant.

## Results

### Target of DEP, DMP, and DOP

We used “diethyl phthalate”, “dimethyl phthalate”, and “dioctyl phthalate” as keywords and screened the relevant targets in the ChEMBL, STITCH, and Swiss Target prediction databases. Using “diethyl phthalate” as the keyword, 10, 8, and 100 relevant targets were obtained from the ChEMBL, STITCH, and Swiss Target Prediction databases, respectively. Using “dimethyl phthalate” as the keyword, 6, 0, and 100 relevant targets were obtained from the ChEMBL, STITCH, and Swiss Target Prediction databases, respectively. Using “dimethyl phthalate” as the keyword, 11, 10, and 100 relevant targets were obtained from the ChEMBL, STITCH, and Swiss Target Prediction databases, respectively. We obtained 243 DEP-, DMP-, and DOP-related targets by integrating and de-weighting the screened targets.

### Target of infertility

The keyword “infertility” was used to screen the Therapeutic Target, OMIM, and Genecard databases. A total of 5,261 relevant targets were identified after integration and deduplication.

### Constructing a PPI network diagram

**FIGURE 2:**
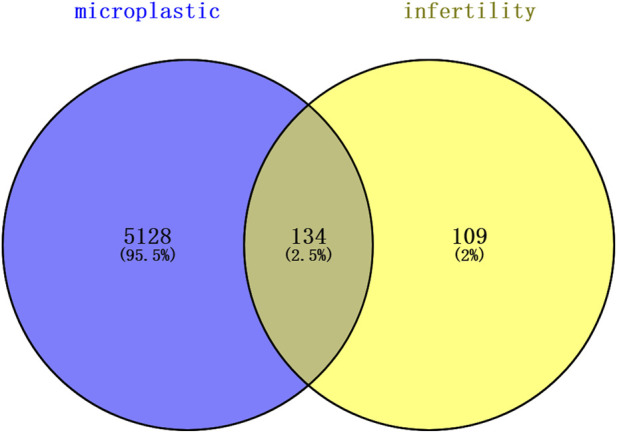
The veen diagram about the target of plasticisers and the target of Infertility. The yellow circle represents the target of plasticisers, and the blue circle represents the target of the intersection of the two circles represents the target of plasticisers for Infertility.

**FIGURE 3:**
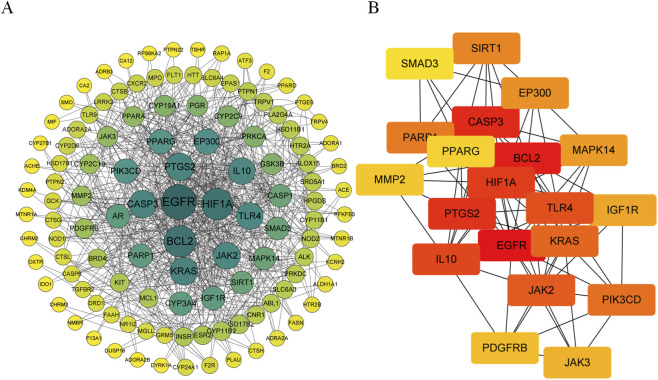
PPI network of plasticisers in the treatment of infertility. **(A)** The nodes represent potential therapeutic targets of plasticisers against infertility. The larger the node, the higher the target degree and the more connections to other nodes. **(B)** Relationship between the hub targets. The redder the color of the node, the higher the target degree and the more connections to other nodes.

Venn diagrams of DEP, DMP, and DOP-related and infertility therapeutic targets (displayed in Figure 2), access to 134 potential targets of DEP, DMP, and DOP for infertility treatment. Potential therapeutic targets were imported into the STRING online platform to obtain PPI network diagrams, which were imported into the Cytoscape software for processing and analysis to obtain PPI network diagrams, and the 20 highest-scoring hub genes were screened (Figure 3).

### GO and KEGG analysis

**FIGURE 4:**
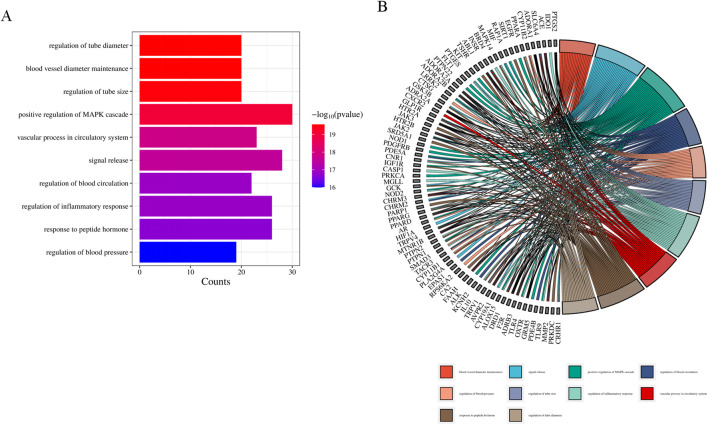
Top ten significant biological process (BP) entries. **(A)** GO enrichment analysis of therapeutic targets for biological process. **(B)** Relationship between the therapeutic targets and biological process.

**FIGURE 5:**
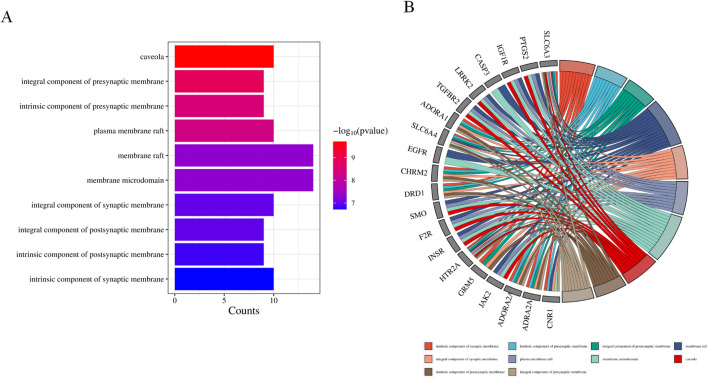
Top ten significant cell component (CC) entries. **(A)** GO enrichment analysis of action targets for cell component. **(B)** Relationship between the action targets and cell component.

**FIGURE 6:**
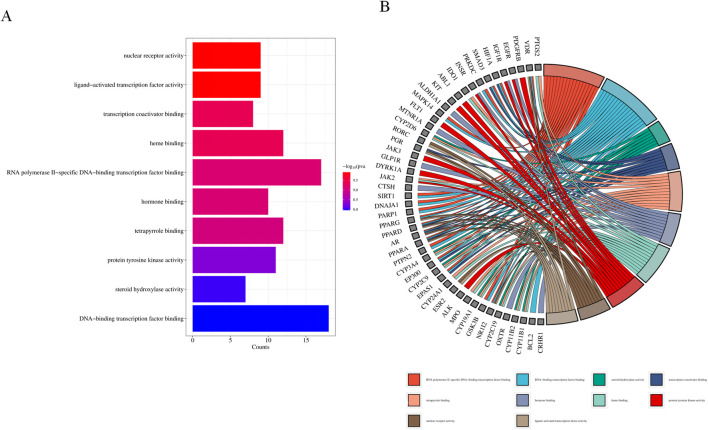
Top ten significant molecular function (MF) entries. **(A)** GO enrichment analysis of action targets for molecular function. **(B)** Relationship between the action targets and molecular function.

**FIGURE 7:**
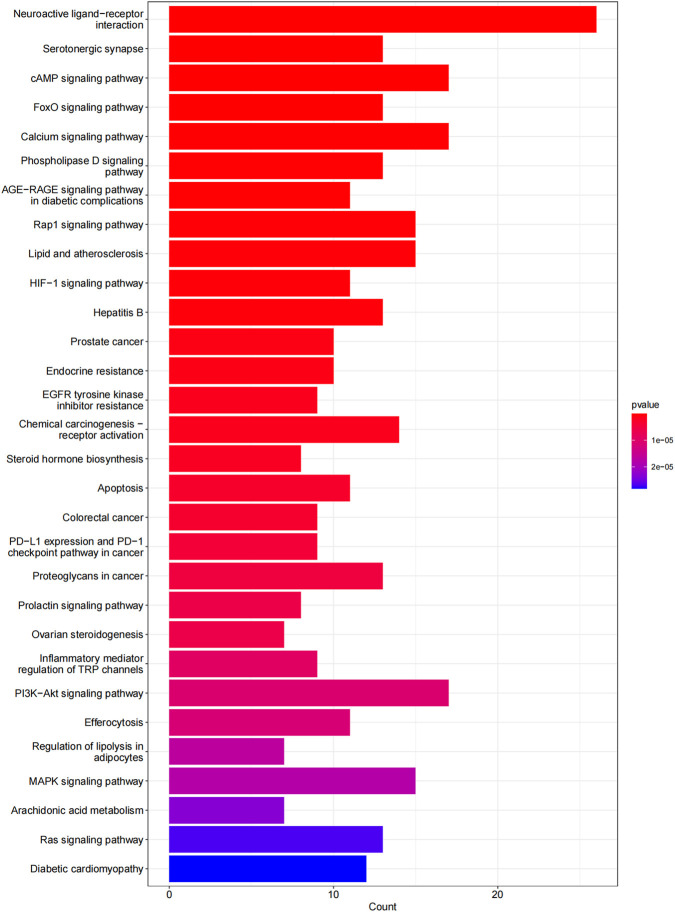
KEGG enrichment analysis for action targets.

The potential targets of DEP, DMP, and DOP for treating infertility were enriched and analyzed using the R-package-Bioconductor Cluster Profiler in R software, and BP, CC, and MF were elaborated on the results after GO enrichment analysis. The top 10 B P, CC, and MF are shown in Figures 4–6, the relationships of the top 10 biological processes and cellular components and all the molecular functions with the potential targets are shown in Figures 4B, 5B, 6B, respectively, respectively. The potential targets of DEP, DMP, and DOP in the affect of infertility were analyzed by KEGG enrichment analysis; the results showed that DEP, DMP, and DOP impacts infertility through multiple signaling pathways. The top 30 signaling pathways are shown in Figure 7.

### Molecular docking simulation

**FIGURE 8:**
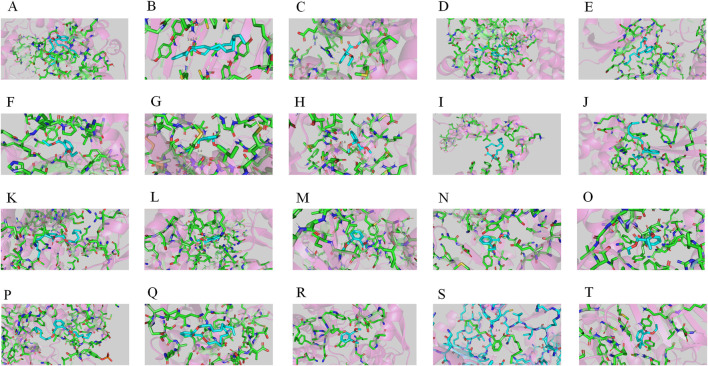
Molecular docking results of plasticisers and hub genes. **(A)** MAPK14, DOP Free Energy: ‐6.6 kcal/mol; **(B)** CASP3, DOP Free Energy: ‐6.4kcal/mol; **(C)** PPARG, DEP Free Energy: ‐6.3 kcal/mol; **(D)** PPARG, DOP Free Energy: ‐6.4kcal/mol; **(E)**: JAK3, DOP Free Energy: ‐6.1 kcal/mol; **(F)** EP300, DEP Free Energy: ‐6.0 kcal/mol; **(G)** (HIF1A, DEP, -5.9kcal/mol); **(H)** (HIF1A, DMP, ‐5.9kcal/mol), **(I)** (IL10, DOP, ‐5.9kcal/mol); **(J)** (EP300, DOP, ‐5.8kcal/mol), **(K)** (PIK3CD, DOP, ‐5.7kcal/mol); **(L)** (SIRT1, DMP, ‐5.7kcal/mol), **(M)** (EGFR, DMP, ‐5.6); **(N)** (PARP1, DEP, ‐5.6kcal/mol); **(O)** (PTGS2, DEP, ‐5.6kcal/mol); **(P)** (JAK2, DOP, ‐5.5kcal/mol); **(Q)** (KRAS, DOP, -7.8kcal/mol); **(R)** (PIK3CD, DMP; ‐5.5kcal/mol); **(S)** (SIRT1, DOP, ‐7.7kcal/mol); **(T)** (CASP3, DEP, ‐7.7kcal/mol).

Molecular docking of hub genes obtained using Autodock software. The molecular docking results were visualized using PyMOL software, and the docking results for the four hub targets are shown in Figure 8. Specific information regarding the docking results is presented in Table 1. Docking of the binding free energy < -4 kcal/mol between one of the targets and the small molecule of DEP, DMP, and DOP indicates that the relevant target can bind freely to DEP, DMP, and DOP.

**TABLE 1:** Information on the docking results of the top 20 significant molecules.

Receptor	Ligands	Free Energy(kcal/mol)	Corresponding serial numbers in Figure 9
MAPK14	DOP	−6.6	A
CASP3	DOP	−6.4	B
PPARG	DEP	−6.3	C
PPARG	DOP	−6.2	D
JAK3	DOP	−6.1	E
EP300	DEP	−6	F
HIF1A	DEP	−5.9	G
HIF1A	DMP	−5.9	H
IL10	DOP	−5.9	I
EP300	DOP	−5.8	J
PIK3CD	DOP	−5.7	K
SIRT1	DMP	−5.7	L
EGFR	DMP	−5.6	M
PARP1	DEP	−5.6	N
PTGS2	DEP	−5.6	O
JAK2	DOP	−5.5	P
KRAS	DOP	−5.5	Q
PIK3CD	DMP	−5.5	R
SIRT1	DOP	−5.5	S
MAPK14	DOP	−6.6	T

### MEHP agents impaired viability of ovarian GCs

**FIGURE 9:**
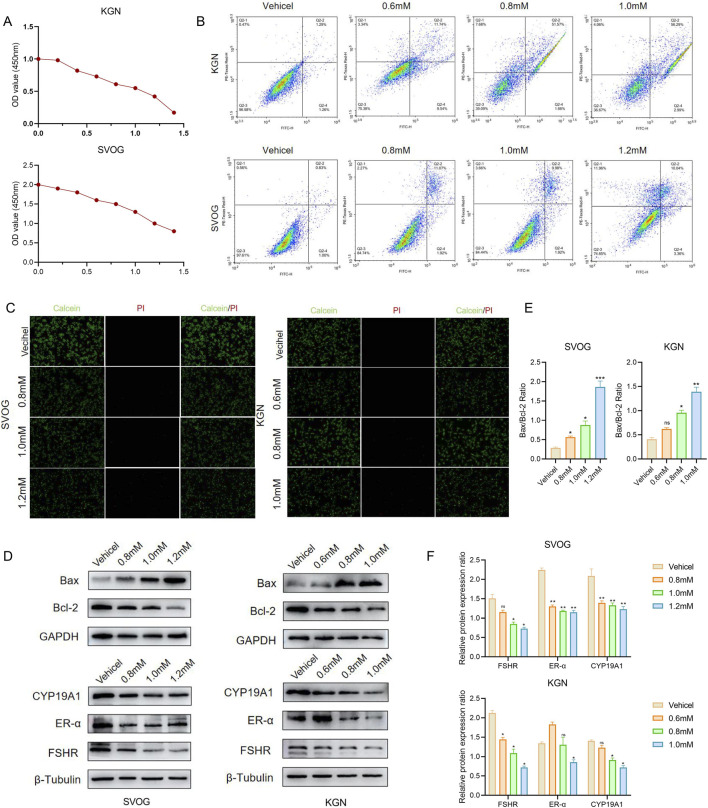
MEHP agents induced ovarian GCs injury. **(A)** CCK-8 assay to measure SVOG and KGN cell viability after MEHP (0, 0.2, 0.4, 0.6, 0.8, 1.0, 1.2, 1.4 mM) treatments for 24 h. **(B)** KGN cells treated with MEHP (0.6, 0.8, 1.0 mM) and SVOG cells treated with MEHP (0.8, 1.0, 1.2 mM) for 24 h and then stained with annexin V-FITC/PI were determined using flow cytometry. **(C)** Live/Dead assay staining for KGN cells with MEHP (0.6, 0.8, 1.0 mM) and SVOG cells with MEHP (0.8, 1.0, 1.2 mM) treatment (green for live cells, red for dead cells). Scale bar = 100 µm. **(D,E)** The protein levels of Bax, Bcl-2, CYP19A1, ER-α, FSHR were examined using Western blot analysis in SVOG and KGN cells treated with MEHP for 24 h. **(F)** Quantitative analysis of the targeted protein expressions. Different letters between groups indicate significant differences at P < 0.05. * P < 0.05, ** P < 0.01, *** P < 0.001, ****P < 0.000.

SVOG and KGN cells were used to detect the effects of MEHP on ovarian function. The results of CCK-8 assays indicated that MEHP treatment markedly reduced cell proliferation in a dose-dependent manner (Figure 9A). Flow cytometry results indicated that MEHP treatment induced apoptosis in SVOG and KGN cells (Figure 9B). Dead/Living staining results and Bax/Bcl-2protein immunoblot assay also confirmed that MEHP treatment induced apoptosis in SVOG and KGN cells in a dose-dependent manner (Figures 9C,D). Furthermore, different concentrations of MEHP significantly downregulated the expression of CYP19A1, ER-α, and FSHR in ovarian GCs, which represents the secretory function of the ovary (Figures 9E,F).

## Discussion

Infertility is estimated to affect 186 million people worldwide (Chen et al., 2020). Although male infertility contributes to more than half of all cases of global childlessness, infertility remains a social burden (Fofie Tedongmo et al., 2024). Human exposure to plasticizers began in the 1930s and has been on the rise ever since, with an increasing number of products containing plasticizers (Cervena et al., 2021). Exposure to these compounds has been detected in food, water, air, consumer products, and biological matrices (urine, blood, feces, meconium, breast milk, amniotic fluid, and saliva), has been associated with impaired human health in fetuses, infants, children, and adults, and has been classified as a priority environmental pollutant because of its ability to interfere with environmental and human health (Cervena et al., 2021). As this exposure has increased, the incidence of infertility has also increased over the past few decades, and the known risk factors are insufficient to explain this increase (Erfani et al., 2019). Therefore, infertility is linked to continued human exposure to plasticizers. In this experiment, the action of plasticizers on infertility and its mechanism were predicted using a network toxicology approach, which showed that it may regulate biological behaviors, such as apoptosis and ovarian steroidogenesis, in infertility through the signal networks formed by multiple signaling pathways for signal interoperability, and the hubs of its therapeutic targets include EGFR, BCL2, CASP3, PTGS2, and HIF1A. IL10, TLR4, JAK2, KRAS, PIK3CD, PARP1, SIRT1, EP300, MAPK14, IGF1R, JAK3, PDGFRB, MMP2, PPARG, SMAD3. Based on the results of network toxicology, we used MEHP to treat SVOG and KGN cells in an *in vitro* experiment to examine its effects on apoptosis, and ovarian steroidogenesis, of the human ovarian granulosa cell lines. The results showed that MEHP inhibited the proliferation of SVOG and KGN cells and promoted apoptosis. The results of flow cytometry indicated that MEHP treatment induced the apoptosis of SVOG and KGN cells, and the dead/living staining results and Bax/Bcl-2 protein immunoblot assay also confirmed that MEHP treatment induced the apoptosis of SVOG and KGN cells in a dose-dependent manner. Furthermore, different concentrations of MEHP significantly downregulated the expression of CYP19A1, ER-α, and FSHR in ovarian GCs, which represents the secretory function of the ovary.

Apoptosis, a common type of cell death, is generated through a complex signaling network that activates the caspase cascade reaction. This includes both intrinsic and extrinsic apoptosis (Funk et al., 2020; Carneiro and El-Deiry, 2020). An increased Bcl-2/BAX ratio and activation of caspase 9 lead to inward apoptosis (Zou et al., 2022; Castaldo et al., 2016). Increased expression of the anti-apoptotic protein BCL-2, decreased apoptotic protein BAX, and increased BCL-2/BAX ratio mediated cell apoptosis (Debatin, 2004; Laulier et al., 2012). In addition, it was demonstrated that activation of the signal networks could lead to apoptosis and inhibit the secretory function of the ovary. The results of this study suggest that plasticizers can regulate inward apoptosis and estrogen secretion in ovarian GCs through a signaling network, leading to infertility. However, this experiment only proved that plasticizers have the effect of inhibiting the growth and promoting the apoptosis of infertility *in vitro*. Whether plasticizers regulate inward apoptosis and estrogen secretion through other signaling pathways and whether they still have the same effect *in vivo* still needs to be further explored.

Although network pharmacology and molecular docking techniques offer the advantages of being cost-effective and highly efficient, the results of these techniques in this study have only been validated through *in vitro* experiments. These findings have not yet been validated through *in vivo* experiments.

## Conclusion

In conclusion, the present study suggests that plasticizers induce apoptosis and estrogen secretion, leading to infertility, which may be achieved by modulating a network of signaling interoperability formed by hub-target connections. Although further studies are needed to fully understand the specific mechanisms by which plasticizers cause infertility, our findings provide a positive foundation.

## Data Availability

The datasets presented in this study can be found in online repositories. The names of the repository/repositories and accession number(s) can be found in the article/supplementary material.

## Glossary

DEP: diethyl phthalate
DMP: dimethyl phthalate
DOP: dioctyl phthalate
EGFR: Epidermal growth factor receptor
CASP3: caspase 3
PTGS2: Prostaglandin G/H synthase 2
HIF1A: Hypoxia-inducible factor 1-alpha
IL10: Interleukin-10
TLR4: Toll-like receptor 4
JAK2: Tyrosine-protein kinase JAK2
KRAS: GTPase KRas
PIK3CD: Phosphatidylinositol 4,5-bisphosphate 3-kinase catalytic subunit delta isoform
PARP1: Poly [ADP-ribose] polymerase 1
SIRT1: NAD-dependent protein deacetylase sirtuin-1
EP300: Histone acetyltransferase p300
MAPK14: Mitogen-activated protein kinase 14
IGF1R: Tyrosine-protein kinase receptor
JAK3: Tyrosine-protein kinase JAK3
PDGFRB: Platelet-derived growth factor receptor beta
MMP2: 72 kDa type IV collagenase
PPARG: Peroxisome proliferator-activated receptor gamma
SMAD3: Mothers against decapentaplegic homolog 3; Apoptosis regulator BAX
BCL-2: Apoptosis regulator Bcl-2
BP: Biological Process
CC: Cellular Component
MF: Molecular Function
GO: Gene Ontology
KEGG: Kyoto Encyclopedia of Genes and Genomes
PPI: Protein-Protein Interaction
CCK8: Cell Counting Kit-8
DMSO: Dimethyl sulfoxide
MEM: Minimum Essential Medium
PI: propidium iodide
FITC: annexin V-fluorescein isothiocyanate
CYP19A1: Aromatase
ER-α: Estrogen Receptor alpha
FSHR: follicle-stimulating hormone receptor
TTD: Therapeutic Target Database
